# Incongruence in Lighting Impairs Face Identification

**DOI:** 10.3389/fpsyg.2022.834806

**Published:** 2022-02-28

**Authors:** Denise Y. Lim, Alan L. F. Lee, Charles C.-F. Or

**Affiliations:** ^1^Division of Psychology, School of Social Sciences, Nanyang Technological University, Singapore, Singapore; ^2^Department of Applied Psychology, Lingnan University, Tuen Mun, Hong Kong SAR, China

**Keywords:** face identification, face recognition, face memory, lighting, mesopic vision, eyewitness testimony

## Abstract

The effect of uniform lighting on face identity processing is little understood, despite its potential influence on our ability to recognize faces. Here, we investigated how changes in uniform lighting level affected face identification performance during face memory tests. Observers were tasked with learning a series of faces, followed by a memory test where observers judged whether the faces presented were studied before or novel. Face stimuli were presented under uniform bright or dim illuminations, and lighting across the face learning and the memory test sessions could be the same (“congruent”) or different (“incongruent”). This led to four experimental conditions: (1) Bright/Dim (learning bright faces, testing on dim faces); (2) Bright/Bright; (3) Dim/Bright; and (4) Dim/Dim. Our results revealed that incongruent lighting levels across sessions (Bright/Dim and Dim/Bright) significantly reduced sensitivity (*d’*) to faces and introduced conservative biases compared to congruent lighting levels (Bright/Bright and Dim/Dim). No significant differences in performance were detected between the congruent lighting conditions (Bright/Bright vs. Dim/Dim) and between the incongruent lighting conditions (Bright/Dim vs. Dim/Bright). Thus, incongruent lighting deteriorated performance in face identification. These findings implied that the level of uniform lighting should be considered in an illumination-specific face representation and potential applications such as eyewitness testimony.

## Introduction

**Figure 1:**
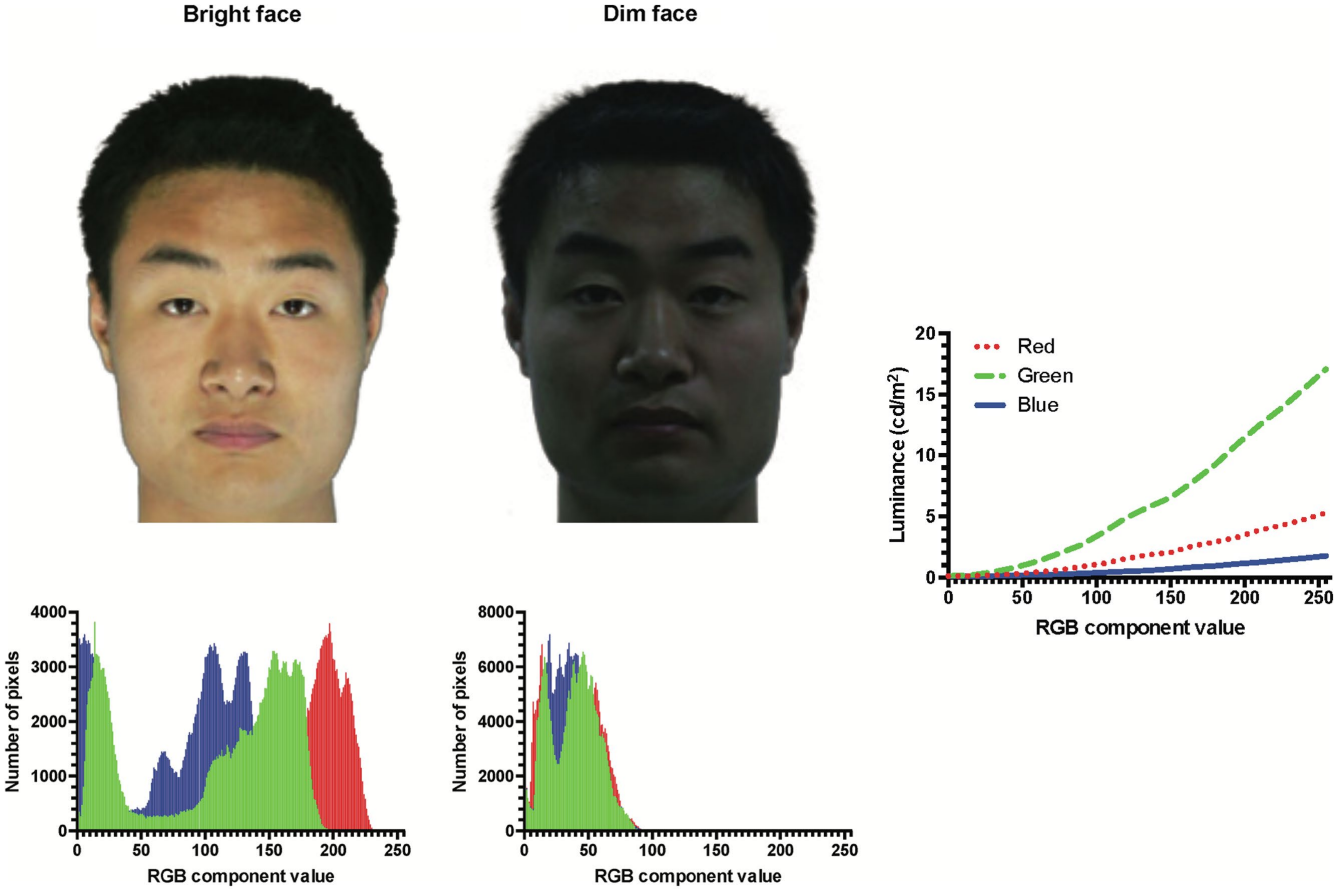
Example faces (top row) of the same identity under bright (left) or dim (right) lighting and their corresponding color histograms (bottom row). In actual experiments, the faces were displayed against a black background in a dark room. The rightmost plot shows the screen luminance as a function of RGB component value measured by a photometer.

Changes in overall lighting introduce broad changes in human visual processing, including visual acuity (e.g., Sheedy et al., 1984; Ferwerda, 1998; Hiraoka et al., 2015), contrast sensitivity (e.g., Amesbury and Schallhorn, 2003; Alghwiri and Whitney, 2012; Wood, 2020), and color appearance (e.g., Shin et al., 2004; Kelber et al., 2017). These may influence our ability to recognize faces, as the level of lighting can affect the amount and/or the type of information gathered from faces (see Figure 1 comparing the same face under uniformly bright and dim lighting).

Despite the potential influence of lighting on face perception, few studies attempted to demonstrate the relationship between levels of uniform lighting and face identification performance. The face memory tests by DiNardo and Rainey (1989) suggested that immediately studied faces were identified with higher accuracy in a brighter room (12 cd/m^2^) than in a dimmer room (6 cd/m^2^), though both luminance levels fell within only a small range under well-lit photopic conditions. Using a broader range of illumination levels (three settings: 0.7 lux—starlight, 10 lux—twilight, 300 lux—office space), Nyman et al. (2019) also found an increase in accuracy with illumination level (together with an increase in confidence and a decrease in response time) in identifying the live-person target from a lineup of face photos under the same light level. Overall, these studies suggested that dimmer light had a generally negative impact on face identification.

One question about lighting’s effect is whether face information encoded under a particular lighting level is generalized so that the encoded face can be recognized at another lighting level, which can appear rather differently (Figure 1). This is an important consideration as faces are often presented under different lighting levels. Imagine that you meet many new friends in an outdoor camp starting midday. You struggle to recognize them during the campfire at night. In broad daylight the next morning, however, you easily recognize your new friends again. This could be a manifestation of the encoding specificity principle, which suggests that better performance is expected when the target at the retrieval stage is presented under the same conditions as the target at the encoding stage (Tulving and Thomson, 1973; Shapiro and Penrod, 1986).

There were few studies on the effect of lighting change on face recognition. DiNardo and Rainey (1991) attempted to systematically investigate how the change in uniform lighting level from encoding to retrieval would affect face identification performance. In that study, face photos were presented at one of two illumination levels (Bright or Dim) with a tachistoscope. Participants were first asked to memorize 10 face photos, all at one illumination level, followed by a memory test of 40 face photos (including the previous 10), either at the same illumination level as during memorization (i.e., congruent lighting: Bright/Bright, or Dim/Dim), or at a different illumination level (i.e., incongruent lighting: Bright/Dim, or Dim/Bright). Contrary to what would be predicted by the encoding specificity principle, DiNardo and Rainey’s (1991) results appeared to suggest that brighter illumination at *any* stage (memorization and/or memory test) led to superior sensitivity *d’* in face identification due to putatively more information available for visual processing. Accordingly, their Bright/Bright condition resulted in the highest *d’* among the four conditions. In addition, they found consistent response biases across all lighting conditions.

However, interpretation of DiNardo and Rainey’s (1991) findings may be complicated by concerns about their methodology. First, both their bright (0.03 foot candles = 0.32 lux) and dim (0.002 foot candles = 0.022 lux) conditions fell within the range of night sky illumination without artificial light. Second, their study might be underpowered with only 10 participants per condition in a between-subjects design to overcome the variability arising from individual differences. Wagenaar and colleagues (Wagenaar and Van Der Schrier, 1996; De Jong et al., 2005) partly addressed these issues with a within-subjects design to assess face identification performance across a broader range of 9 illumination levels (0.3 to 3,000 lux simulating full moon night through cloudy daylight illumination) during face learning. Their results suggested a general increase in performance with illumination level at encoding. However, they only used one illumination level (“normal lighting”) at the retrieval stage, thus only data under the equivalence of Bright/Bright and Dim/Bright conditions were available but data under Bright/Dim and Dim/Dim conditions were lacking. Hence, potential effects from the lighting change between encoding and retrieval should be further understood.

In this study, we seek to examine lighting’s effects on face identity processing in an experiment about learning of a series of face images followed by a face memory test. Here, we focused on two lighting levels (photopic/mesopic), using face images photographed under uniform bright or dim lighting, respectively (Figure 1). The lighting level was kept consistent within any learning or test session. The major questions to address are: (1) How would face identification performance be affected by lighting level when it remained congruent across the learning and test sessions (i.e., Bright/Bright vs. Dim/Dim)? (2) How would changes in lighting level from learning to test (i.e., incongruent lighting conditions: Bright/Dim and Dim/Bright) influence face identification performance compared to congruent lighting levels across sessions (Bright/Bright and Dim/Dim)? We adopted a within-subjects design so that each observer participated in all four lighting conditions (Bright/Dim, Bright/Bright, Dim/Bright, and Dim/Dim) of the face memory experiment.

## Materials and Methods

### Participants

A total of 24 Chinese observers (14 females, mean age = 22.4 years, age range: 19–26 years, 21 from Singapore and 1 each from Malaysia, Hong Kong, and mainland China) with extensive prior exposure to Chinese faces participated in the experiment. All observers were naïve to the purpose of study, had normal, or corrected-to-normal, visual acuity, provided written informed consent and received honoraria or course credits for their participation, as approved by the Psychology Ethics Committee of Nanyang Technological University in accordance with the Declaration of Helsinki. None of the observers were familiar with the face stimuli prior to experiment.

### Stimulus Display

The experiment was presented using a Desktop computer running Psychopy version 3.2.4. Stimuli were displayed on a monitor at a refresh rate of 120 Hz, with a screen resolution of 1,920 × 1,080 pixels and a color depth of 24 bits/pixel placed at a viewing distance of 70 cm (pixel size: 0.023°) in a dark experimental room with no light source other than the monitor screen. The monitor settings were adjusted in order to display the dimmest possible range of screen luminance (from 0.09 cd/m^2^ for pure black screen to 23.61 cd/m^2^ for pure white screen, as measured by a Minolta LS-100 photometer). The screen luminances of component values ranging from 0 to 255 were also measured separately for the red, green, and blue (RGB) channels (Figure 1, rightmost plot).

### Stimuli

Face images of 128 male Chinese individuals posed at full-front views with neutral expressions were retrieved with permission from the Oriental Face Database created by the Institute of Artificial Intelligence and Robotics of Xi’an Jiaotong University.^1^ Each individual was photographed twice (example in Figure 1): once under bright lighting, and another under dim lighting; both in color with uniform illumination in a purposely constructed photography room. The bright and dim light settings were consistent across individuals. The head sizes were rescaled to subtend a standardized height of 15.13° (crown to chin) and the widths ranging from 10.46°–12.58° (mean: 11.5°). No alterations were made to the images except replacing the background by uniform black, so that variations in local color, luminance and contrast were faithful to the source images. Facial features (e.g., blemishes) that could potentially serve as salient cues in identification were removed. When displayed on our screen, the stimulus luminance ranged from 4.39–9.46 cd/m^2^ (mean ± 1 *SD*: 7.07 ± 0.97 cd/m^2^; within the photopic range) for brightly lit faces, and from 0.60–2.12 cd/m^2^ (mean ± 1 *SD*: 1.13 ± 0.25 cd/m^2^; within the mesopic range) for dimly lit faces.

### Procedure

#### Lighting Conditions

The experiment consisted of four conditions, each started with learning a series of faces followed by a face memory test. The four conditions corresponded to different combinations of lighting during learning and testing: (1) Bright/Dim, that is, learning brightly lit faces, then testing on dimly lit faces, (2) Bright/Bright, (3) Dim/Bright, and (4) Dim/Dim. Prior to the experiment, observers were dark-adapted in the experimental room for 10 min. Then, they performed the four conditions in an order determined by the complete counterbalancing design (thus 24 observers fulfilled all possible permutations).

#### Face Learning

In each condition, observers were first instructed to passively view and remember 16 faces of different identities that they were told would later appear in the memory test among other new faces. All faces were photographed under the same lighting conditions (i.e., faces were either all brightly lit, or all dimly lit). The 16 faces were presented randomly in sequence (once per face) and then repeated immediately in a second, differently randomized sequence. Thus, there were a total of 32 face presentations (16 faces × 2 repetitions) in the face learning session. Each face presentation was preceded by a white fixation cross (size: 0.97 × 0.97°) centrally presented against a uniform black screen, which vanished after 500 ms and was replaced by a centrally presented face for 2000 ms. Thus, each face was displayed for a total of 4,000 ms across two repetitions. These setups had been verified by pilot experiments to optimize face learning.

#### Face Memory Test

Immediately after face learning, observers performed a face memory test to identify the 16 faces just learnt (“studied faces”) from 16 new ones (“distractor faces”). The 32 test faces (always under the same lighting among themselves) were presented in a random sequence. Each test face was presented only once for 350 ms at the center of screen, preceded by a 500-ms central fixation (as in face learning). The 350 ms test duration is common in face recognition research and is long enough for one to two eye fixations, which have been shown to be sufficient for face identification (Hsiao and Cottrell, 2008). Observers were instructed to maintain central fixation when the face was presented and to press one of two keys to indicate whether the face was studied before, or considered novel, as promptly and as accurately as possible after stimulus offset. No feedback was provided concerning the correctness of responses. The next trial initiated automatically following response to the previous trial. Observers were given a 3-min break between conditions, during which they remained seated in the dark room. None of the 32 face identities presented in one condition would be used again in any other conditions (note that there were 128 distinct face identities in our database; see “Stimuli”). In both learning and memory test sessions, head sizes were randomly jittered between 80 and 120% of the original sizes to avoid faces potentially memorized based on low-level image features (see “Discussion”). The entire experiment lasted approximately 45 min.

### Data Analysis

For each observer, performance was analyzed in the form of sensitivity *d’*:


(1) $$d\prime =z\left(H\right)-z\left(F\right)$$

where H is the hit rate, that is, proportion of studied faces correctly identified in the memory test, and F is the false alarm rate, that is, proportion of distractor faces incorrectly identified as studied during face learning.

Response bias was calculated in the form of criterion *c*:


(2) c=−[z(H)+z(F)]/2

To avoid infinite values of *d’* and *c*, *H* = 1 was converted to *H* = 1–1/(2*n_s_*), *n_s_* = 16 (number of studied face trials), and *F* = 0 to *F* = 1/(2*n_d_*), *n_d_* = 16 (number of distractor face trials), as recommended by Macmillan and Creelman (2005).

The hit rates, false alarm rates, sensitivities *d’* and criteria *c* from 24 observers were analyzed and compared across conditions. Where a repeated measures ANOVA was performed, the Holm–Bonferroni correction (Holm, 1979) was applied for post-hoc multiple comparisons.

## Results

### Hit Rate

Hit rates (Table 1) were compared across the four conditions using a one-way repeated measures ANOVA. Mauchly’s test of sphericity indicated that the sphericity assumption was met, *χ*^2^(5) = 10.452, *p* = 0.064. There was a significant main effect of lighting condition, *F*(3, 69) = 14.506, *p* < 0.001, $\eta_{p}^{2}$ = 0.387. Post-hoc multiple comparisons (Table 2) indicated that congruent lighting across the face learning and the memory test sessions (Bright/Bright and Dim/Dim) led to significantly more hits than incongruent lighting across sessions (Bright/Dim and Dim/Bright).

**Table 1:** Means and SEMs of hit rates and false alarm rates for all four conditions.

Condition	Hit rate		False alarm rate	
	*Mean*	*SEM*	*Mean*	*SEM*
Bright/Dim	0.560	0.035	0.266	0.034
Bright/Bright	0.776	0.031	0.250	0.030
Dim/Bright	0.625	0.040	0.227	0.030
Dim/Dim	0.750	0.032	0.216	0.036

**Table 2:** Pairwise differences in hit rates and sensitivities *d’* between conditions and their effect sizes (Cohen’s *d*).

Pairwise comparisons between conditions	Hit rate			Sensitivity *d*’		
	*Difference*	*p*	*Cohen’s d*	*Difference*	*p*	*Cohen’s d*
Bright/Bright—Bright/Dim	0.216	<0.001^*^	1.099	0.713	<0.001^*^	0.921
Bright/Bright—Dim/Bright	0.151	0.004^*^	0.761	0.375	0.010^*^	0.667
Dim/Dim—Bright/Dim	0.190	<0.001^*^	1.452	0.813	<0.001^*^	1.334
Dim/Dim—Dim/Bright	0.125	0.005^*^	0.723	0.475	<0.001^*^	1.029
Bright/Bright—Dim/Dim	0.026	0.485	0.145	0.100	0.218	0.259
Dim/Bright—Bright/Dim	0.065	0.346	0.287	0.338	0.077	0.448

The values of *p* were Holm–Bonferroni corrected.

* *p* < 0.05.

### False Alarm Rate

The false alarm rates, with means ranging from 0.216 to 0.266 across the four lighting conditions (Table 1), were analyzed using a one-way repeated measures ANOVA. Mauchly’s test of sphericity indicated that the sphericity assumption was met, *χ*^2^(5) = 1.250, *p* = 0.940. There was no significant main effect of lighting condition, *F*(3, 69) = 0.968, *p* = 0.413, $\eta_{p}^{2}$ = 0.040, indicating no significant differences in false alarm rates across conditions.

### Sensitivity *d’*

**Figure 2:**
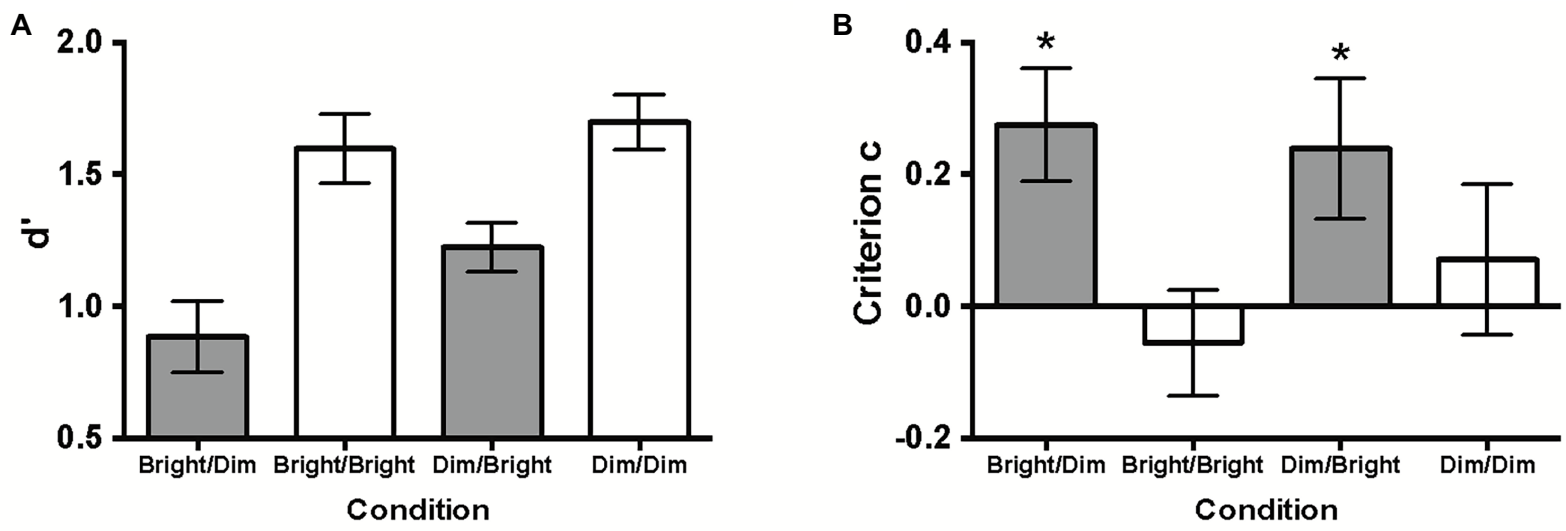
**(A)** Sensitivity *d’* and **(B)** criterion *c* (indicating response bias) across the four conditions. All error bars represent ±1 SEM. The asterisks (^*^) in **(B)** represent *c* significantly different from zero. Conservative response biases were found in both incongruent lighting conditions (Bright/Dim & Dim/Bright).

Observers’ face identification performances were analyzed in terms of sensitivity *d’* (Figure 2A). Separate two-tailed one-sample t-tests showed that the *d’* values for all four lighting conditions deviated significantly from zero (*p*s < 0.001), indicating that observers identified the studied faces in all conditions.

These *d’* values were then compared across conditions using a one-way repeated measures ANOVA. Mauchly’s test of sphericity indicated that the sphericity assumption was violated, *χ*^2^(5) = 17.698, *p* = 0.003; thus, the Greenhouse–Geisser correction was used. There was a significant main effect of lighting condition, *F*(2.108, 48.476) = 17.962, *p* < 0.001, $\eta_{p}^{2}$ = 0.438.

Similar to hit rate comparisons, post-hoc multiple comparisons on *d’* (Table 2) also indicated that congruent lighting (Bright/Bright and Dim/Dim) led to significantly better face identification than incongruent lighting (Bright/Dim and Dim/Bright).

### Response Bias (Criterion *c*)

We additionally measured potential response biases in the four conditions in terms of criterion *c* (Figure 2B). Separate two-tailed one-sample t-tests revealed that the *c* values were significantly different from zero in both incongruent lighting conditions (Bright/Dim: *t*(23) = 3.224, *p* = 0.004, Cohen’s *d* = 0.658; Dim/Bright: *t*(23) = 2.252, *p* = 0.034, Cohen’s *d* = 0.460). The positive *c* values indicated conservative response biases (i.e., observers inclined to label any faces as unseen) in these conditions. Additionally, a paired samples t-test showed that the *c* values between Bright/Dim and Dim/Bright were not significantly different, *t*(23) = 0.354, *p* = 0.726 (two-tailed), Cohen’s *d* = 0.072.

However, *c* was not significantly different from zero in either congruent lighting condition (Bright/Bright: *t*(23) = −0.685, *p* = 0.500, Cohen’s *d* = −0.140; Dim/Dim: *t*(23) = 0.625, *p* = 0.538, Cohen’s *d* = 0.128), implying no evidence of response biases. Thus, observers responded conservatively only under incongruent lighting conditions.

## Discussion

We examined the effects of uniform lighting level on face identity processing in a face memory experiment. Observers’ sensitivity values (*d’*) demonstrated significantly above chance performance in face identification among all conditions, regardless of lighting levels during the learning and memory test sessions. Importantly, *d’* varied across the four lighting conditions consistent with the encoding specificity principle in memory studies (Tulving and Thomson, 1973; Shapiro and Penrod, 1986). When the lighting levels were congruent across sessions (Bright/Bright and Dim/Dim), the *d’* values were consistently higher than when lighting was changed, or incongruent, across sessions (Bright/Dim and Dim/Bright).

In terms of the decision criteria *c*, we found more conservative response biases in the incongruent lighting conditions where observers had a tendency to indicate faces as unseen, but we did not detect any evidence of response biases in either congruent lighting condition. The conservative criterion shifts (and drops in *d’*) were solely driven by the significantly lower hit rates in the incongruent lighting conditions than in the congruent lighting conditions, whereas the false alarm rates were comparable across all conditions. In other words, observers were better at correctly identifying studied faces but not better at rejecting distractor faces under congruent lighting than under incongruent lighting. Notably, such results agreed with the Neyman–Pearson objective of maintaining a false alarm rate (Neyman and Pearson, 1933; Green and Swets, 1966) for placing a conservative bias when sensitivity dropped under incongruent lighting conditions.

These results did not demonstrate any mirror effect (Glanzer and Adams, 1985, 1990), where better recognition performance would be accompanied by both a higher hit rate and a lower false alarm rate. Rather, our results were consistent with a dual-factor account of recognition memory (Hockley et al., 1999; Vokey and Hockley, 2012), suggesting that false alarm rate was determined by general familiarity of the stimulus class, while hit rate was determined by recollection (retrieval of specific details from prior experience). We may reasonably argue that both bright and dim faces are familiar to us; hence, comparable false alarm rates. In contrast, recollection of studied faces was impaired by incongruent lighting, leading to lower hit rates.

We found an effect of incongruent lighting, which was not reported in DiNardo and Rainey (1991). Such a difference may be explained by the higher statistical power in the present study, using a larger sample size and a more sensitive within-subjects design. Our findings were consistent with those by Wagenaar and colleagues (Wagenaar and Van Der Schrier, 1996; De Jong et al., 2005), though neither of their studies included all four lighting conditions as we did here (see “Introduction”).

Despite how differences in lighting would lead to changes in many aspects of the visual appearance of faces, it was assumed that humans could cope with such changes by generating a more abstract visual representation of the face, which is generalized across lighting variations (e.g., structural codes for faces, see Bruce and Young, 1986). This assumption is also behind classic object recognition models (e.g., Marr and Nishihara, 1978; Biederman, 1987; Biederman and Ju, 1988), proposing that the visual system extracts illumination invariant features such as edges and contours for object recognition.

However, many studies on the effects of illumination *direction* on face recognition (e.g., Hill and Bruce, 1996; Gauthier and Tarr, 1997; Tarr et al., 1998; Braje, 2003; etc.) suggested otherwise. In particular, an image-based model with illumination directions encoded in face representations was proposed. This was because changes in illumination direction, which introduced complex changes in shading gradient, shadow, and overall magnitude of illumination, often led to poorer performance in face recognition.

It is important to note, however, that lighting in those studies was applied *non-uniformly* to faces by varying the direction of illumination, while the present study manipulated the level of *uniform* lighting. Our uniform-lighting manipulations did not introduce the kind of shading or shadow changes otherwise created from a different illumination direction. Thus, the performance cost we novelly found due to uniform-lighting changes suggests that uniform lighting, in addition to non-uniform lighting, is probably also encoded in illumination-sensitive face representations. When lighting was changed from encoding to retrieval, the illumination-specific facial information encoded might not be readily available for use in identifying faces at a different lighting level, leading to a decline in performance. It would be interesting to examine these potentially illumination-specific face representations in future studies.

A potential explanation for superior performance under congruent lighting could be partly due to the use of identical face photos across sessions (except varying in size), allowing for low-level pattern matching (Hancock et al., 2000) besides high-level face identity matching. In contrast, the different face photos in incongruent lighting conditions might not permit pattern matching due to lighting and other superficial image differences. Thus, we randomly varied the face size across presentations, a control common for reducing the possibility of pattern matching (Webster and MacLeod, 2011). Notably, face size variation modifies brain response to otherwise same-size identical faces (Grill-Spector et al., 1999; Andrews and Ewbank, 2004), though it does not impair face identity processing (e.g., Zhao and Chubb, 2001; Yamashita et al., 2005; Jeffery et al., 2006; Lee et al., 2006). Nevertheless, the present findings might be verified by future experiments using different photos of the same face across sessions in all lighting conditions.

Our results failed to show significant differences in sensitivity between the two congruent lighting conditions, Bright/Bright and Dim/Dim. However, prior studies (DiNardo and Rainey, 1989, 1991; Nyman et al., 2019) suggested a general decline in face identification performance with dimmer lighting, following observations that dim lighting reduces the amount of available information that can be extracted from images (e.g., scenes, digit arrays, as in Loftus, 1985). The apparent inconsistency between these data and ours may be explained by the different ranges of luminance/illuminance levels studied. Although bright faces and dim faces appeared differently in our study, the facial features remained visible at both luminance levels, despite one under photopic vision and another under mesopic vision. It is possible that sensitivity (and criterion) for face identification remain constant over the range of lighting levels we tested, even crossing the photopic/mesopic boundary. Facial information available under bright lighting may not be identical to facial information available under dim lighting, but the divergent information can be processed equally effectively. It would be interesting to identify the potential diversity of diagnostic facial features available at different levels of lighting. Nevertheless, performance in face identification may generally increase with lighting level over a wider range (e.g., from very dark starlight to much brighter office space, as suggested by Nyman et al., 2019). Further studies should be designed to systematically examine the effect of a broader range of lighting levels on face recognition. After all, it should be noted that our sample size (*N* = 24) was less sensitive statistically to detect a small, but real, difference (e.g., required effect size = 0.49 for achieving a high power of 1−*β* = 0.95 with *ρ* = 0.80 (empirical correlation in our sample) and *α* = 0.05; Cohen, 1988).

The direction of change in lighting conditions from learning to memory test (Bright/Dim vs. Dim/Bright) did not cause significant changes in face identification performance. This implies that the transfer of diagnostic information from encoding to retrieval deteriorates to a similar degree from bright to dim as from dim to bright. We did not detect any advantage when bright faces were initially encoded, consistent with the result that our dim lighting settings did not reduce sensitivity to faces (*cf.* similar performances for Dim/Dim and Bright/Bright conditions).

Our study was among the few that demonstrated the effects of uniform lighting on face identity processing, using images of real faces photographed under bright and dim uniform lighting presented within the photopic and mesopic ranges, respectively. There are a few questions to be addressed in future studies. First, it remains unclear whether diagnostic features visible in faces differ across levels of lighting, and, if so, how they may contribute to illumination-specific face representations in the brain. Also, it would be interesting to examine how such representations might become more generalized if observers studied the same faces under a wider range of lighting levels and different directions of illumination. Another pending question would be how much dimmer the lighting would need to be before face identification performance declines, given that the level of dim lighting in this study resulted in comparable performance as under bright lighting. Finally, it remains unclear how high-level face identification is connected to low-level vision under dim lighting, which involves complex interactions between rod and cone activations that are not well understood in mesopic vision (see, for review, Buck, 2004, 2014; Zele and Cao, 2015). For example, dim faces may appear more blue than bright faces due to additional rod inputs (Purkinje shift: Shin et al., 2004; Kelber et al., 2017).

While the present findings highlight the need to understand uniform lighting as a factor underlying face representation, they are also relevant to practical needs such as security and eyewitness testimony, where inaccurate face recognition could lead to detrimental outcomes. While many crimes happen at night, police lineups are usually performed in brightly illuminated rooms in police stations. The lighting mismatch may possibly reduce the eyewitness’s sensitivity to faces and increase bias to report faces as unseen, with errors potentially leading to wrongful convictions. Thus, it is important to further understand the impact of lighting on face recognition for both theoretical and practical purposes.

## Data Availability Statement

The raw data supporting the conclusions of this article will be made available by the authors, without undue reservation.

## Ethics Statement

The studies involving human participants were reviewed and approved by Psychology Ethics Committee, Nanyang Technological University. The participants provided their written informed consent to participate in this study.

## Author Contributions

DL and CO contributed to conception and design of the study and wrote the first draft of the manuscript. DL conducted the experiment. DL, AL, and CO performed the statistical analysis. AL wrote sections of the manuscript. All authors contributed to manuscript revision, read, and approved the submitted version.

## Funding

This work was supported by NTU HASS Start-Up Grant and Singapore MOE AcRF Tier 1 Grant 2018-T1-001-069 and 2019-T1-001-064 to CO, and 2019-T1-001-060 to CO and AL. DL was a recipient of the SGUnited Traineeships Programme.

## Conflict of Interest

The authors declare that the research was conducted in the absence of any commercial or financial relationships that could be construed as a potential conflict of interest.

## Publisher’s Note

All claims expressed in this article are solely those of the authors and do not necessarily represent those of their affiliated organizations, or those of the publisher, the editors and the reviewers. Any product that may be evaluated in this article, or claim that may be made by its manufacturer, is not guaranteed or endorsed by the publisher.

## Footnotes

^1^ http://gr.xjtu.edu.cn/web/jianyi/english-version

## References

[ref1] Alghwiri A. A. Whitney S. L. (2012). “Balance and falls,” in Geriatric Physical Therapy. 3rd Edn. eds. Guccione A. Wong R. Avers D. (St. Louis: Mosby), 331–353.

[ref2] Amesbury E. C. Schallhorn S. C. (2003). Contrast sensitivity and limits of vision. Int. Ophthalmol. Clin. 43, 31–42. doi: 10.1097/00004397-200343020-00006 12711901

[ref3] Andrews T. J. Ewbank M. P. (2004). Distinct representations for facial identity and changeable aspects of faces in the human temporal lobe. NeuroImage 23, 905–913. doi: 10.1016/j.neuroimage.2004.07.060, PMID: 15528090

[ref4] Biederman I. (1987). Recognition-by-components: A theory of human image understanding. Psychol. Rev. 94, 115–147. doi: 10.1037/0033-295X.94.2.115, PMID: 3575582

[ref5] Biederman I. Ju G. (1988). Surface versus edge-based determinants of visual recognition. Cogn. Psychol. 20, 38–64. doi: 10.1016/0010-0285(88)90024-2, PMID: 3338267

[ref6] Braje W. L. (2003). Illumination encoding in face recognition: effect of position shift. J. Vis. 3, 4–170. doi: 10.1167/3.2.4, PMID: 12678618

[ref7] Bruce V. Young A. (1986). Understanding face recognition. Br. J. Psychol. 77, 305–327. doi: 10.1111/j.2044-8295.1986.tb02199.x 3756376

[ref8] Buck S. L. (2004). “Rod-cone interactions in human vision,” in The Visual Neurosciences. eds. Chalupa L. M. Werner J. (Cambridge, MA: MIT Press), 863–878.

[ref9] Buck S. L. (2014). “The interaction of rod and cone signals: pathways and psychophysics,” in The New Visual Neurosciences. eds. Chalupa L. M. Werner J. (Cambridge, MA: MIT Press), 485–499.

[ref10] Cohen J. (1988). Statistical Power Analysis for the Behavioral Sciences. 2nd *Edn*. New York: Lawrence Erlbaum Associates.

[ref11] De Jong M. Wagenaar W. A. Wolters G. Verstijnen I. M. (2005). Familiar face recognition as a function of distance and illumination: a practical tool for use in the courtroom. Psychol. Crime Law 11, 87–97. doi: 10.1080/10683160410001715123

[ref12] DiNardo L. Rainey D. (1989). Recognizing faces in bright and dim light. Percept. Mot. Skills 68, 836–838. doi: 10.2466/pms.1989.68.3.836 2748299

[ref13] DiNardo L. Rainey D. (1991). The effects of illumination level and exposure time on facial recognition. Psychol. Rec. 41, 329–334. doi: 10.1007/BF03395115

[ref14] Ferwerda J. A. (1998). Fundamentals of spatial vision. Applic. Visual Percept. Comput. Graphics 140, 1–27.

[ref15] Gauthier I. Tarr M. J. (1997). Becoming a “Greeble” expert: exploring mechanisms for face recognition. Vis. Res. 37, 1673–1682. doi: 10.1016/S0042-6989(96)00286-6, PMID: 9231232

[ref16] Glanzer M. Adams J. K. (1985). The mirror effect in recognition memory. Mem. Cogn. 13, 8–20. doi: 10.3758/bf03198438 4010518

[ref17] Glanzer M. Adams J. K. (1990). The Mirror effect in recognition memory: data and theory. J. Exp. Psychol. Learn. Mem. Cogn. 16, 5–16. doi: 10.1037/0278-7393.16.1.5, PMID: 2136752

[ref18] Green D. M. Swets J. A. (1966). Signal Detection Theory and Psychophysics. New York: Wiley.

[ref19] Grill-Spector K. Kushnir T. Edelman S. Avidan G. Itzchak Y. Malach R. (1999). Differential processing of objects under various viewing conditions in the human lateral occipital complex. Neuron 24, 187–203. doi: 10.1016/S0896-6273(00)80832-6, PMID: 10677037

[ref20] Hancock P. J. B. Bruce V. Mike Burton A. (2000). Recognition of unfamiliar faces. Trends Cogn. Sci. 4, 330–337. doi: 10.1016/S1364-6613(00)01519-9 10962614

[ref21] Hill H. Bruce V. (1996). Effects of lighting on the perception of facial surfaces. J. Exp. Psychol. Hum. Percept. Perform. 22, 986–1004. doi: 10.1037//0096-1523.22.4.986, PMID: 8756964

[ref22] Hiraoka T. Hoshi S. Okamoto Y. Okamoto F. Oshika T. (2015). Mesopic functional visual acuity in Normal subjects. PLoS One 10:134505. doi: 10.1371/journal.pone.0134505, PMID: 26218066 PMC4517889

[ref23] Hockley W. E. Hemsworth D. H. Consoli A. (1999). Shades of the mirror effect: recognition of faces with and without sunglasses. Mem. Cogn. 27, 128–138. doi: 10.3758/BF03201219, PMID: 10087862

[ref24] Holm S. (1979). A simple sequentially rejective multiple test procedure. Scand. J. Stat. 6, 65–70. doi: 10.2307/4615733

[ref25] Hsiao J. H.-W. Cottrell G. (2008). Two fixations suffice in face recognition. Psychol. Sci. 19, 998–1006. doi: 10.1111/j.1467-9280.2008.02191.x, PMID: 19000210 PMC7360057

[ref26] Jeffery L. Rhodes G. Busey T. (2006). View-specific coding of face shape. Psychol. Sci. 17, 501–505. doi: 10.1111/j.1467-9280.2006.01735.x, PMID: 16771800

[ref27] Kelber A. Yovanovich C. Olsson P. (2017). Thresholds and noise limitations of colour vision in dim light. Philos. Trans. R. Soc. B Biol. Sci. 372:20160065. doi: 10.1098/rstb.2016.0065, PMID: 28193810 PMC5312015

[ref28] Lee Y. Matsumiya K. Wilson H. R. (2006). Size-invariant but viewpoint-dependent representation of faces. Vis. Res. 46, 1901–1910. doi: 10.1016/j.visres.2005.12.008, PMID: 16469348

[ref29] Loftus G. R. (1985). Picture perception: effects of luminance on available information and information-extraction rate. J. Exp. Psychol. Gen. 114, 342–356. doi: 10.1037/0096-3445.114.3.342, PMID: 3161980

[ref30] Macmillan N. A. Creelman C. D. (2005). Detection Theory: A User’s Guide. 2nd Edn. Mahwah: Psychology Press.

[ref31] Marr D. Nishihara H. K. (1978). Representation and recognition of the spatial organization of three-dimensional shapes. *Proceedings of the Royal Society of London*. Series B Biol. Sci. 200, 269–294. doi: 10.1098/rspb.1978.0020, PMID: 24223

[ref32] Neyman J. Pearson E. S. (1933). On the problem of the most efficient tests of statistical hypotheses. *Philosophical transactions of the Royal Society of London*. Series A Contan.Papers Math. Physic Char. 231, 289–337.

[ref33] Nyman T. J. Antfolk J. Lampinen J. M. Tuomisto M. Kaakinen J. K. Korkman J.. (2019). A stab in the dark: The distance threshold of target identification in low light. Cogent Psychol. 6:2047. doi: 10.1080/23311908.2019.1632047

[ref34] Shapiro P. N. Penrod S. (1986). Meta-analysis of facial identification studies. Psychol. Bull. 100, 139–156. doi: 10.1037/0033-2909.100.2.139

[ref35] Sheedy J. E. Bailey I. L. Raasch T. W. (1984). Visual acuity and chart luminance. Am. J. Opto. Vision Sci. 61, 595–600. doi: 10.1097/00006324-198409000-00010 6507580

[ref36] Shin J. C. Yaguchi H. Shioiri S. (2004). Change of color appearance in Photopic, Mesopic and Scotopic vision. Opt. Rev. 11, 265–271. doi: 10.1007/s10043-004-0265-2

[ref37] Tarr M. J. Kersten D. Bülthoff H. H. (1998). Why the visual recognition system might encode the effects of illumination. Vis. Res. 38, 2259–2275. doi: 10.1016/S0042-6989(98)00041-8, PMID: 9797998

[ref38] Tulving E. Thomson D. M. (1973). Encoding specificity and retrieval processes in episodic memory. Psychol. Rev. 80, 352–373. doi: 10.1037/h0020071

[ref39] Vokey J. R. Hockley W. E. (2012). Unmasking a shady mirror effect: recognition of normal versus obscured faces. Q. J. Exp. Psychol. 65, 739–759. doi: 10.1080/17470218.2011.628399, PMID: 22182292

[ref40] Wagenaar W. A. Van Der Schrier J. H. (1996). Face recognition as a function of distance and illumination: A practical tool for use in the courtroom. Psychol. Crime Law 2, 321–332. doi: 10.1080/10683169608409787

[ref41] Webster M. A. MacLeod D. I. A. (2011). Visual adaptation and face perception. Philos. Trans. R. Soc. B Biol. Sci. 366, 1702–1725. doi: 10.1098/rstb.2010.0360, PMID: 21536555 PMC3130378

[ref42] Wood J. M. (2020). Nighttime driving: visual, lighting and visibility challenges. Ophthalmic Physiol. Opt. 40, 187–201. doi: 10.1111/opo.12659, PMID: 31875993

[ref43] Yamashita J. A. Hardy J. L. De Valois K. K. Webster M. A. (2005). Stimulus selectivity of figural aftereffects for faces. J. Exp. Psychol. Hum. Percept. Perform. 31, 420–437. doi: 10.1037/0096-1523.31.3.420, PMID: 15982123

[ref44] Zele A. J. Cao D. (2015). Vision under mesopic and scotopic illumination. Front. Psychol. 5:1594. doi: 10.3389/fpsyg.2014.01594, PMID: 25657632 PMC4302711

[ref45] Zhao L. Chubb C. (2001). The size-tuning of the face-distortion after-effect. Vis. Res. 41, 2979–2994. doi: 10.1016/S0042-6989(01)00202-4, PMID: 11704237

